# Combination of DNA-hypomethylating agent and hematopoietic stem cell transplantation in treatment of juvenile myelomonocytic leukemia: A case report

**DOI:** 10.1097/MD.0000000000023606

**Published:** 2020-12-11

**Authors:** Yuan Ai, Xiaoxi Lu, Tingting Zhu, Yiping Zhu, Hanmin Liu, Shuwen Sun

**Affiliations:** a Department of Pediatrics, West China Second University Hospital, Sichuan University; b Key Laboratory of Obstetric & Gynecologic and Pediatric Diseases and Birth Defects of Ministry of Education, Sichuan University, Chengdu, Sichuan, China.

**Keywords:** allogenic hematopoietic stem cell transplantation, DNA-hypomethylating agents, juvenile myelomonocytic leukemia

## Abstract

**Introduction:**

Juvenile myelomonocytic leukemia (JMML) is a rare myeloproliferative neoplasm of early childhood characterized by excessive proliferation of myelomonocytic cells and an aggressive clinical course. Allogenic hematopoietic stem cell transplantation (HSCT) is a firmly established treatment, but patients without fully matched donors have poor prognoses. Disease recurrence is the main cause of treatment failure. Meanwhile, most cases with splenomegaly present with platelet transfusion refractoriness, but splenectomy remains controversial. DNA hypermethylation correlates with poor prognosis in JMML; however, hypomethylating therapy alone does not eradicate leukemic clones. Thus, a suitable treatment with a good success rate remains elusive.

**Patient concerns:**

Here, we report our experience with a patient who suffered from recurrent fever, pallor, abdominal distention, leukocytosis, and thrombocytopenia with a silent past history and family history of somatic *KRAS* mutation. The patient was treated with decitabine as a bridging therapy before haploidentical HSCT. Decitabine was also used prophylactically after transplantation.

**Diagnosis:**

We arrived at a JMML diagnosis after observing leukocytosis, less than 20% blast cells in the peripheral blood and bone marrow, increased monocyte counts, negativity for the BCR-ABL fusion gene, positivity for somatic *KRAS* mutation, and massive splenomegaly.

**Interventions:**

The patient accepted splenectomy before HSCT, and haploidentical HSCT was applied after treatment with a DNA-hypomethylating agent. The hypomethylating agent was administered for 1 year after HSCT to prevent disease recurrence.

**Outcomes:**

The patient presented with complete remission of the disease and mild graft versus host disease for 26 months after treatment with decitabine and HSCT.

**Lessons:**

Combining haploidentical HSCT and DNA-hypomethylating agents may improve the prognosis of JMML. Meanwhile, splenectomy could be an effective option in cases with massive splenomegaly and platelet transfusion refractoriness.

## 1 Introduction

Juvenile myelomonocytic leukemia (JMML) is a rare hematopoietic disorder of infancy/early childhood and is an overlap of myelodysplastic and myeloproliferative neoplasms.^[1,2,3]^ The incidence of JMML is reported to be 0.6 to 1.2 cases per million children per year, and JMML accounts for 1% to 3% of pediatric leukemia cases.^[2–7]^ Most cases are diagnosed before 6 years of age, with a median age of 1.8 years, and 35% of the patients are younger than 1 year of age at presentation. JMML can occasionally occur in newborns.^[6,8,9]^ Approximately 90% of patients carry either somatic or germline mutations of *PTPN-11*, *KRAS*, *NRAS*, *CBL*, or *NF1* in their leukemic cells, although other classic genetic disorders, such as monosomy 7, have been reported.^[5,10,11]^ These genetic aberrations all activate the RAS/mitogen-activated kinase signaling pathway, which results in the hypersensitivity of JMML progenitors to granulocyte-macrophage colony-stimulating factor.^[10]^ Whole-exome strategies have also identified secondary mutations in *SETBP1* and *JAK3* in up to 17% of JMML patients, and these mutations are reported to confer a poorer prognosis.^[12]^

According to the International JMML Working Group criteria, it is not difficult to confirm a diagnosis of JMML.^[6,10,11]^ The clinical manifestation of JMML can be somewhat nonspecific, but a diagnosis must include all of the following criteria: splenomegaly, an absolute monocyte count >1000/μL, higher fetal hemoglobin levels than normal for the age of the patient, <20% myeloblasts in the blood and bone marrow, and the absence of a *t* (9;22) or BCR/ABL fusion gene.

While hematopoietic stem cell transplantation (HSCT) remains the only curative treatment option for JMML, disease relapse after therapy occurs in as many as 40% to 50% of cases.^[8]^ An increasing number of studies show that both DNA hypermethylation and acute myeloid leukemia transformation before HSCT in JMML generally lead to poor outcomes.^[8,10,11]^ Thus far, most reports describing the use of a hypomethylating agents before HSCT have focused on azacytidine, while relatively few have examined decitabine for JMML treatment. Here we report on a patient with JMML and a *KRAS* somatic mutation who was given a pre-transplant splenectomy and a course of the hypomethylating agent decitabine as a bridge to transplantation with a favorable result.

## 2 Methods

This study retrospectively analyzed the clinical data of a patient with JMML and a *KRAS* mutation who was admitted between 2016 and 2017 in the West China Second University Hospital and followed up on for 26 months. This study was approved by the Ethics Committee of the West China Second University Hospital, and written informed consent was obtained from the parents of the child. The patient was followed up on for 26 months, and is still alive and in complete remission of the disease with mild graft-versus-host disease (GVHD) of the skin.

## 3 Case report

A 10-month-old male infant who presented with recurrent fever, pallor, and abdominal distention was admitted to the Division of Pediatric Hematology and Oncology of West China 2nd University Hospital. There was no significant family history of hematologic disease, and on clinical examination he was found to have massive splenomegaly, mild hepatomegaly, and cervical lymphadenopathy. A complete blood count showed leukocytosis, anemia, thrombocytopenia, and that myeloblasts accounted for 1% of all nucleated cells, as shown in Table 1. Examination of bone marrow aspirate revealed that it was hypercellular with granulocytic proliferation and a decreased ratio of megakaryocytes, while myeloblasts were not substantially increased in number. Analysis of a peripheral blood sample revealed negativity for the BCR/ABL fusion gene, while a molecular study investigating genes often shown to be aberrant in JMML showed a mutation in somatic *KRAS*.

**Table 1:** Complete blood count of the patient before treatment.

	Cell counts (× 10^9^/L)	Ratio (%)	Normal reference range (× 10^9^/L)
White blood cells (WBCs)	14.3		5.7–13.2
Neutrophils	2.43	16.9	0.5–4.91
Metamyelocytes	0.29	2	
Metarubricytes	0.4	2.7	
Hemoglobin	55 g/L		102–146 g/L
Platelets	8		151–536
Myeloblast cells		1%	

The patient received 2 courses of protocol contained homoharringtonine and cytarabine (homoharringtonine 2.5 mg/m^2^ for 7 consecutive days, cytarabine 40 mg/m^2^ for 7 consecutive days) and 3 courses of a decitabine protocol (20 mg/m^2^ over 1 hour intravenously (i.v.) for 5 consecutive days, repeated every 4 weeks). The patient suffered from platelet transfusion refractoriness, and was then transferred to the pediatric surgery department, where a splenectomy was performed. There were no complications after the operation, and approximately 2 weeks later he had a good response to platelet transfusion. Eleven months after the diagnosis, the patient underwent haploidentical stem cell transplantation from his mother, with a 4-human-leukocyte-antigen (HLA)-mismatched umbilical cord stem cell sample used for complementary therapy. The conditioning regimen administered to the patient was myeloablative, including busulfan (4.8 mg/kg/d i.v. for 4 days), cyclophosphamide (60 mg/kg/d for 2 days), F-ATG (5 mg/kg/d i.v. for 3 days), and melphalan (140 mg/m^2^/d i.v. for 1 day). The numbers of CD34+ cells and monocytes infused during transplantation were 5.0 × 10^6^/kg and 6.6 × 10^8^/kg, respectively, while the umbilical cord blood stem cells infused were 1.65 × 10^5^/kg of CD34+ cells and 7.2 × 10^7^/kg of monocytes. The prophylaxis to combat GVHD included methotrexate (15 mg/m^2^ on day 1 and 10 mg/m^2^ on days 3, 5, and 11), cyclosporine (3 mg/kg/d during the conditioning regimen), and mycophenolate mofetil (250 mg orally bid from day 1 to day 44). Neutrophil engraftment was noted on day 14, with platelet engraftment seen on day 16. On day 20, the patient's total blood cells were 99.73% donor cells, and by day 30, a test to detect the *KRAS* mutation was negative. One month after HSCT, the patient developed grade I GVHD of the skin on day 58 in the form of an erythematous rash, which responded to oral methyl-prednisolone (4 mg orally bid). Two months after the transplant, we administered decitabine prophylactically (20 mg/m^2^ over 1 hour i.v. once a month) to guard against disease relapse. Twenty-six months after transplantation, the patient's blood tests were normal, the molecular genetic analyses were negative for mutations, and complete chimerism was observed.

## 4 Discussion

The median survival time of JMML patients who do not receive an allograft is only 10 to 12 months. Most untreated patients die from respiratory failure due to pulmonary infiltration by leukemic cells or progression to acute myeloid leukemia. Intensive chemotherapy is largely unsuccessful in JMML because of an increased risk of treatment-related mortality, a low rate of true remission, and a long-term survival of less than 10%. Currently, only HSCT has been proven to result in long-term remission of the disease.^[5,9,13,14]^

In the majority of studies published so far describing the use of HSCT in JMML, the authors enrolled a limited number of patients treated with heterogeneous approaches. In a European Working Group on Childhood MDS (EWOG-MDS)/European Blood and Marrow Transplantation (EBMT) trial in which 100 JMML patients received HSCT, the authors reveal that the 5-year survival after transplantation was 64%, while the 5-year event-free survival (EFS) was 52%. Conditioning regimens using busulfan, cyclophosphamide, and melphalan were coincident, and all the transplants from an unrelated donor were either identical or had 1 antigenic/allelic disparity. The myeloablative conditioning chemotherapy, which included busulfan, was associated with a better EFS and a lower relapse incidence. Avoiding total-body irradiation (TBI) can reduce radiation-induced complications without increasing the incidence of relapse. Another clinical study from EUROCORD, EBMT, EWOG-MDS, and the Center for International Blood and Marrow Transplant Research retrospectively analyzed 110 JMML patients given unrelated donor umbilical cord blood transplantations. The 1 and 2 to 3 HLA antigen disparities were 43% and 35%, respectively, and the 5-year EFS and overall survival rates were 44% and 52%, respectively.^[15]^ These results suggest that the TBI performed during the conditioning regimen was associated with a greater probability of hematopoietic recovery.^[15]^ The lack of an appropriate HLA-compatible donor for our patient, together with his youth (he was younger than 2 years old), meant that TBI was not a good choice in this case, so we chose to employ haploidentical HSCT with the patient's mother as a donor. We also chose a myeloablative conditioning regimen (which consisted of busulfan and melphalan) to protect against disease recurrence, with cyclosporine A, F-ATG, and low-dosage methotrexate selected for GVHD prophylaxis. The EUROCORD–Center for International Blood and Marrow Transplant Research study shows that grades II–III of acute GVHD were associated with a decreased incidence of relapse, while other studies have reported that the development of chronic GVHD may be associated with better survival.^[4,11]^ Our patient presented with both acute and chronic GVHD, which we hope may play a role in preventing relapse of the disease.

Massive splenomegaly may be associated with high tumor burden or platelet transfusion refractoriness; however, performing a pre-transplant splenectomy as well as the relevance of spleen size itself at the time of allograft for patients with JMML is controversial. The EWOG-MDS/EBMT JMML trial demonstrated that patients with JMML who have different spleen sizes (>5 cm, <5 cm) at HSCT and post-splenectomy at HSCT showed no statistical difference in EFS, relapse incidence, or transplantation-related mortality. However, other studies have noted a trend toward an EFS benefit after splenectomy (56% vs 36%; *P* = .098).^[11,13]^ In the current case report, the reasons for the splenectomy were specific. The patient was younger than 18 months old and showed platelet transfusion refractoriness. After the splenectomy, he was routinely given benzathine penicillin for 6 months to prevent infection, and he suffered no severe infections before the transplantation. Our experience with this case suggests that for a younger patient with platelet transfusion refractoriness, a pre-transplantation splenectomy could be an effective choice to reduce disease-associated mortality.

While HSCT remains the only curative treatment option for JMML, the main problem with this therapy is disease relapse, which can occur in as many as 40% to 50% of cases.^[8]^ Since 2011, several studies have reported DNA hypermethylation at various genetic loci, which is involved in epigenetic alterations in JMML. Aberrant methylation was shown to be largely confined to CpG island regions and correlated with a worse prognosis.^[8]^ Based on these data, hypomethylating therapy for the treatment of JMML was proposed in 2009. It has been reported that a patient with JMML, a *KRAS* mutation, and monosomy 7 was treated with azacytidine (i.v. on 5 consecutive days, repeated every 4 weeks) and displayed an impressive clinical and hematologic response, with the monosomy 7 phenotype disappearing at the sixth cycle.^[16]^ Due to the high incidence of relapse, some physicians have advocated the prophylactic use of hypomethylating agents over the course of the 1st year after HSCT in order to prevent recurrent attacks.^[8]^ The clinical and hematological responses of the patient were observed after 4 cycles of chemotherapy that included cytarabine, homoharringtonine, and decitabine. After HSCT, he was treated with decitabine (20 mg/m^2^ over 1 hour i.v. once a week) for 1 year to prevent malignancy recurrence. Twenty-six months after HSCT, the patient remains disease-free. Our results suggest that post-transplant use of hypomethylating agents may be an effective regimen for guarding against relapse.

No risk-stratification system for patients with JMML has been described to date. Low platelet count (<33 × 109/L), age above 2 years at diagnosis, and high fetal hemoglobin levels ≥10% at diagnosis have been reported as the main predictors of short survival.^[10,11,14]^

For patients with JMML presenting with massive splenomegaly, a pre-transplant splenectomy combined with decitabine bridging to HSCT may play an important role in curing the disease. If there is no fully matched related or unrelated donor and the patient is in need of an urgent allograft, complementary transplantation (unrelated cord blood following haploidentical stem cells) may be a suitable choice. In addition, prophylactic use of decitabine after HSCT may also contribute to continuous complete remission of the disease.

## Acknowledgments

The authors thank the patient's parents who provided their information.

## Author contributions

**Conceptualization:** Yuan Ai.

**Data curation:** Yuan Ai, Xiaoxi Lu.

**Funding acquisition:** Xiaoxi Lu.

**Investigation:** Yuan Ai.

**Project administration:** Shuwen Sun.

**Supervision:** Yiping Zhu, Hanmin Liu.

**Writing – original draft:** Yuan Ai, Xiaoxi Lu, Tingting Zhu.

**Writing – review & editing:** Yiping Zhu, Hanmin Liu.
